# Flax Composites With Improved Interfacial Strength Through Microbially Induced Mineral Precipitation

**DOI:** 10.1002/adma.74543

**Published:** 2026-08-08

**Authors:** Deniz Sayinbas, Ingo Nettersheim, Jeong‐Joo Oh, Marie‐Eve Aubin‐Tam, Julie Teuwen, Kunal Masania

**Affiliations:** ^1^ Shaping Matter Lab Faculty of Aerospace Engineering Delft University of Technology Delft Netherlands; ^2^ Department of Bionanoscience, Kavli Institute of Nanoscience Faculty of Applied Sciences Delft University of Technology Delft Netherlands; ^3^ Aerospace Structures and Materials Faculty of Aerospace Engineering Delft University of Technology Delft Netherlands

**Keywords:** bio‐composites, bio‐inspired materials, biomineralization, flax fiber composites, microbially induced calcite precipitation (MICP)

## Abstract

Driven by the needs of modern transportation and the clean energy transition, the demand for sustainable and lightweight materials is increasing. Composite materials incorporating natural fibers such as flax fibers have gained attention due to their carbon‐capturing potential and good specific mechanical properties. However, when embedded in hydrophobic polymer matrices, flax fibers exhibit inferior mechanical performance primarily due to their hydrophilic composition and discontinuous fiber architecture. Biological materials such as nacre have developed useful strategies through mineralization to distribute localized stresses and develop extrinsic toughness that could inspire a solution to enhance stress transfer in natural fiber composites. Here, we report a biomineralization strategy to introduce an additional hierarchy to flax composites. By tuning salt concentrations in the process, we achieve controlled deposition of microbe‐mediated mineral particles on flax yarns. With controlled biomineralization, we show that the minerals can enhance the compressive toughness by 178% and compressive strength by 30%. The findings highlight a novel bio‐inspired pathway for tailoring composite performance through sustainable processing, offering a scalable and environmentally friendly approach to enhance natural fiber composites for structural applications.

## Graphical abstract

A bio‐inspired biomineralization strategy introduces an additional hierarchy to flax fiber composites. By controlling microbe‐mediated mineral particle deposition through tuned salt concentrations, stress transfer within the natural fiber composite is enhanced. This sustainable approach dramatically improves compressive toughness by 178% and compressive strength by 29%, offering an eco‐friendly pathway to advanced, sustainable structural materials.

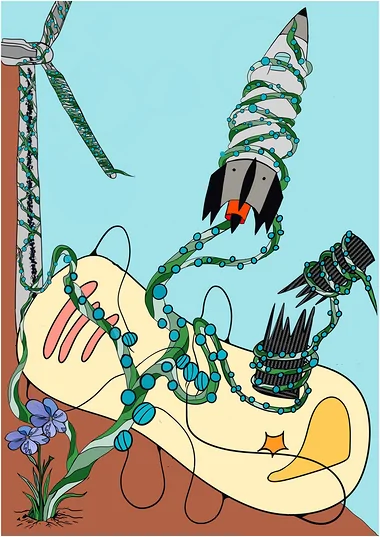

## 1 Introduction

There is a growing need for sustainable, high‐performance, lightweight materials to meet demands for transportation and the clean energy transition [1, 2, 3]. Composite materials consisting of wood [4, 5] or natural fibers could offer potential solutions [6, 7, 8]. From these materials, flax fibers have emerged as viable reinforcing fibers due to their carbon‐capturing potential (1.39 kg.CO_2_/kg) and high specific material properties, which are comparable to aluminum and glass fiber composite materials [9]. Flax fibers achieve their mechnical performance through a highly ordered, cellulose‐rich cell wall with a characteristically low microfibril angle that enables efficient load transfer within a lightweight hierarchical structure [10]. However, due to their biological origin, grown inside flax stems, these fibers are usually finite in length with a maximum length of around 1 m [11], which requires further processing to achieve materials for engineering structures.

To produce continuous‐like fibers for structures, these elementary fibers are usually wet spun together to form continuous long yarns with twist angles in the range 10°–15° [12]. This twist serves an important function in mechanically binding the fibers together. Whilst these materials perform well in tension, a combination of this spun macrostructure and the biopolymeric origin results in poor compression performance [13, 14], which hinders their broader application potential in critical structures.

In addition to their fiber structures, when producing composites with polymeric matrices, the interfacial adhesion between the fibers and polymer is often limited by the surface chemistry of the flax fibers. Flax fibers exhibit poor adhesion to hydrophobic polymer matrices because their surface is rich in hydrophilic cellulose and hemicellulose, leading to weak interfacial bonding and reduced composite strength [15]. This structure‐property relationship of the cellulosic fiber origin and interfacial compatibility often yields low compressive strength, which has been a key limiting material feature of applications such as wind turbine blades [14]. Other microstructural features, such as microfibril angle [16, 17], hollow lumen morphology [18, 19], and waxy residue on the fiber surface [13, 14], also contribute to micro‐buckling and kink‐band formation under compression. The weak shear resistance of the cell wall and the natural defects along the fiber bundles, which occur during flax growth, further promote instability at lower stresses [13, 14, 20].

A wide range of strategies has been explored to enhance fiber–matrix compatibility in natural fiber composites, including physical [21, 22, 23], chemical [24, 25, 26] treatment methods and the grafting of nanoscale reinforcements onto fiber surfaces [27, 28, 29]. Although these approaches can improve interfacial performance, their long‐term effectiveness and scalability are frequently constrained by durability issues, environmental burdens, and performance–sustainability trade‐offs [21]. Despite the mechanical benefits demonstrated by these methods, the reliance on harsh chemistries or non‐biodegradable additives highlights a critical need for more sustainable and scalable material solutions in next‐generation natural fiber composite development.

Nature endows organisms with remarkable strength and toughness by arranging constituents into hierarchical, multiscale architectures into unified, damage‐resistant structures. For example, in nacre, microscopic “brick‐and‐mortar” plates are interlinked by microscopic mineral bridges and organic layers that deflect cracks, enable mechanical binding of platelets, and dissipate energy through crack path tortuosity and interfacial sliding [30, 31, 32, 33]. Wood similarly distributes stress through its anisotropic fiber network that facilitates load transfer across cellular boundaries, and transverse ray fibers, both enhancing resilience [34, 35]. These design strategies might be a promising blueprint for future engineered material systems [36]. We hypothesize that leveraging mineral bridging to create interlinked fiber networks could yield improved performance in natural fiber composites.

Natural materials often gain strength not by being uniformly stiff, but by integrating reinforcement in clever ways that bind softer organic frameworks. Certain bacterial species could provide a promising route to local reinforcement through microbially induced calcite precipitation (MICP). For example, *Sporosarcina pasteurii* induce an alkaline environment through ureolytic activity that elevates pH and supplies carbonate ions, enabling calcium ions in the environment to crystallize and nucleate on a substrate as carbonates. Such bacteria have been used to repair cracks in heritage stone by precipitating calcium carbonate in situ, effectively bridging gaps and restoring structural integrity [37]. In construction, microorganisms precipitate calcium carbonate to seal concrete fissures or consolidate sandy ground, creating durable, self‐healing materials [38, 39, 40]. Moreover, such biomineralization (BM) improves the durability of bacterial cellulose by forming layered composites where mineral bridges fortify the structure [41]. These examples point to a broader potential to tune and reinforce organic substrates in a controlled, sustainable way, opening new directions for material design.

We propose that through bacteria‐mediated precipitation of carbonates on flax fibers, the fibers can be bridged by the mineral particles, forming well‐interlinked fibers within a yarn. Unlike conventional flax fiber treatments, which primarily modify surface chemistry or apply external particle coatings, this approach employs in situ BM, where minerals nucleate directly on fiber functional groups. The property enhancements are driven by multiple mechanisms, including inter‐yarn and intra‐yarn particle bridging as well as an increase in fiber surface roughness, which together promote mechanical interlocking and strengthen fiber–matrix adhesion. The contribution of each mechanism is quantified, enabling a deeper understanding of how microstructural modifications translate into macroscopic property improvements.

## 2 Results and Discussion

Flax fibers consist of distinct layers that are hierarchically structured fibers on different length scales (Figure 1). For many composite applications, semi‐crystalline organized concentric layers of cellulose form technical fibers, which are combined fiber cells called elementary fibers. During the extraction and production of these materials, kinks can be formed that damage their structure and, in combination with the yarn architecture and material chemistry, contribute to weak compression performance. To address this problem, we employ specific conditions where dolomite‐like (magnesium calcite) particles are deposited on fiber surfaces through the activity of *S. pasteurii*. The subsequent precipitation creates a network of nucleated particles that bridge adjacent fibers (inter‐yarn bridging) and reinforce internal yarn structure (intra‐yarn bridging), while simultaneously roughening the fiber surface. These mechanisms collectively contribute to improved compressive behavior and interfacial strength in flax/epoxy composites. Figure 1 presents a detailed overview of the structure of the flax fibers on the left. Critical mechanisms that contribute to the buckling of flax before treatment and the mechanisms that contribute to the compressive and interfacial strength of the flax composites after treatment are shown at the right. Through BM, we hypothesize improved adhesion, interlaminar shear strength, and compressive strength due to roughening of the fibers and particle bridging at the intra‐yarn and inter‐yarn levels. Each feature relies on a different degree of mineral deposition, so the biomineralization process requires careful tuning.

**FIGURE 1:**
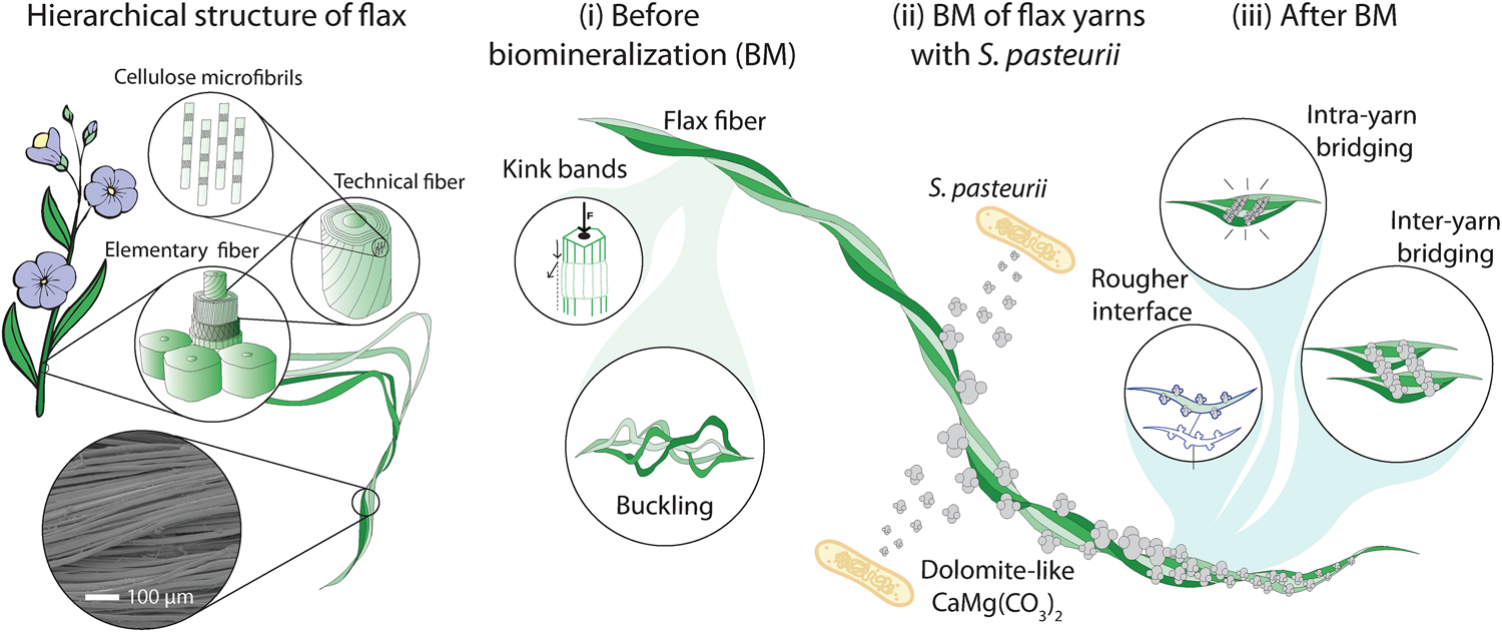
Overview of the hierarchical structure of flax fibers, and the reinforcement strategy via biomineralization (BM). Flax plant and a hierarchical structure of lignocellulosic flax fibers. SEM image of the flax yarns made from elementary flax fibers. Before treatment, yarns made of flax fibers containing growth‐ and processing‐induced kink bands initiate buckling under compression.

To achieve a controlled deposition, various parameters of the broth and bacterial growth conditions must be established. Figure 2a presents the procedure and underlying mechanisms of BM treatment on flax fibers. The flax yarns were incubated in a solution containing *S. pasteurii* for 24 h, boiled for 10 min to deactivate the bacteria, and later dried at 70°C for 3 h in a vacuum oven. Three key parameters during the process were indicated as temperature, salt concentration, and stirring.

**FIGURE 2:**
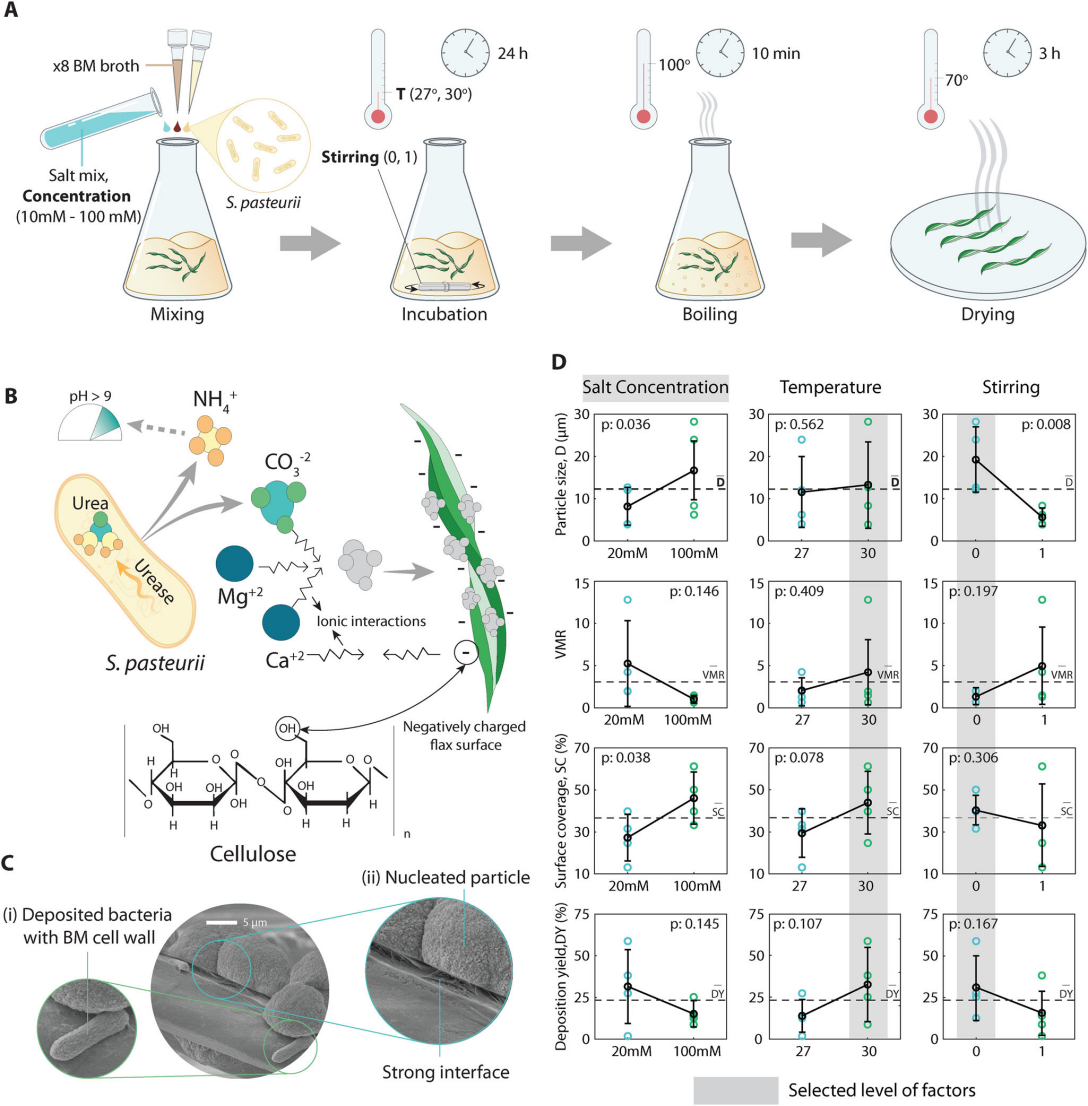
Particle nucleation results in strong adhesion between fibers and the particles. Varying the salt concentration, temperature and stirring during incubation affects particle size, clustering, surface coverage on the fibers and the particle deposition yield. (a) The steps followed to BM of flax yarns indicating the key parameters that can influence the particle nucleation and growth. (b) Urease activity from *Sporosarcina pasteurii* results in decomposition of urea into carbonates and ammonia. Key ionic interactions facilitate the formation of the dolomite‐like particles and nucleation of the particles on the flax that is negatively charged due to ─OH groups on its cellulosic surface. (c) SEM images highlighting 2 types of deposition: (i) through bacteria sticking to the fiber and (ii) through direct particle nucleation. The particle peeled off fibers results in cellulose fibrillation, indicating a strong interface between fiber and the particle. (d) Effects of salt concentration, temperature and stirring on the particle size, variance to mean ratio (VMR), surface coverage and deposition yield. Black circles indicate the mean of each level of factors with their standard deviations. The steeper the black line, the more dominant the effect that the corresponding factor has. The black dashed line indicates the average of the associated plot. *p*‐values for each experiment are given on each corresponding plot.

As shown in Figure 2b, during incubation, *S. pasteurii* produces urease, which catalyzes the breakdown of urea into ammonium and carbonate ions. The release of free ammonium raises the pH, creating an alkaline environment that favors nucleation and crystal growth by enhancing carbonate availability [42, 43]. Positively charged magnesium and calcium ions are attracted to the negatively charged flax surface due to the presence of hydroxyl (─OH) groups on the cellulose. These OH groups serve as nucleation sites for the mineral particles [41, 44]. Consequently, carbonate ions bind with the adsorbed magnesium and calcium ions to form dolomite‐like particles, which precipitate onto the fiber surface. This process resulted in a good interfacial formation between fibers and minerals.

SEM analysis reveals a promising interface between the fibers and precipitated particles, as shown in Figure 2c. This interaction between the fibers and the particles is important to obtain the benefit of mechanical interlocking between the polymer and the particle‐deposited fibers due to the roughened fiber surface. The minerals are not only deposited on the fibers but also on the bacterial cell wall, where bacteria can also stick to the fibers, leading to local precipitation. The observation that the fiber tears or peels while minerals remain suggests that dolomite‐like crystals are effectively “cemented” to the fiber surface. This is likely due to mechanical interlocking within the fiber's microstructure or via extracellular polymeric substances (EPS) produced during BM [45]. The minerals grow directly onto fiber surfaces, conforming to microfibril and lumen structure. This creates mechanical anchoring that resists physical removal far better than spherical or *ex situ* precipitates [46, 47]. Furthermore, the fact that inter‐fiber shear occurs before the particle‐fiber interface fails implies that the interface strength exceeds the cohesive strength of the fiber itself.

To optimize this “cementation process”, the effects of salt concentration, incubation temperature, and stirring on the particle size, particle homogeneity, surface coverage of the particles and deposition yield were investigated using a screening Design of Experiments (DoE) approach (Figure 2d). Increasing the salt concentration in the treatment solution may lead to higher supersaturation, which promotes both nucleation and particle growth, thereby enhancing surface coverage due to the greater availability of ionic species. Elevated temperatures further modulate deposition efficiency by increasing bacterial metabolic activity, resulting in different urease production patterns and accelerated or decelerated urea hydrolysis [48]. This, in turn, facilitates more rapid dolomite‐like precipitation. In contrast, stirring introduces shear forces and fluid disturbances that interfere with nucleation and particle growth, ultimately reducing particle size and deposition yield. The salt concentration can be used as a mechanism for microstructural tuning. Besides, setting the temperature at 30°C and keeping the solution stationary during incubation will yield a uniform deposition and consistent particle size. We identify two variables for each to perform a prospective design of experiments exploration as shown in Figure 2d.

An unreplicated factorial design was evaluated under the assumption of effect sparsity, with experimental error estimated from inactive factors to calculate screening *p*‐values, as also suggested in the literature [49, 50]. The factorial design allows for parameter screening to identify the most influential biomineralization conditions. A *p*‐value smaller than or close to 0.1 shows statistical significance and a strong relationship between the associated factor on the effect. Here, surface coverage represents how well the fibers are covered with minerals, whereas deposition yield represents the efficiency of the deposition with respect to the expected dolomite‐like formation, depending on the amount of salts added into the solution. Surface coverage ultimately determines the roughening of the surface, and deposition yield helps determine whether the BM takes place effectively. Variance to mean ratio (VMR) here is calculated using the quadrant count method to represent the randomness, clustering, or homogeneity of the particle distribution. A VMR > 1 corresponds to clustering, and the clustering increases as VMR gets larger, where a VMR = 1 means homogeneous and a VMR < 1 means a random distribution of particles (Figures S1 and S2). To assess the repeatability of the dominant screening trend and the associated analysis, independent repeat experiments were performed for salt concentration. VMR, particle size, and surface coverage were evaluated and are reported for three independent experimental repeats (Figure S3). Higher salt concentration results in larger particles with less clustering and higher surface coverage on fibers, even though the deposition yield is lower. Temperature has a lower impact on particle size and clustering compared to salt concentration. However, the deposition yield and the surface coverage both increase with higher temperature. We found that the effect of stirring is most dominant on particle size, as it leads to smaller particles.

The parameters that yield the best coverage and deposition yield were determined, and salt concentration was decided to be further investigated. A temperature of 30°C was used, and stirring was avoided during incubation to maximize the deposition yield and surface coverage, as well as to increase the consistency of the process and the particle size. Next, we evaluated the chosen parameters to study the structure‐morphology relationships within the dried and biomineralized flax fiber yarns with different salt concentrations during incubation.

The microstructure can be further modified by changing the salt concentration of the treatment, as shown in Figure 3. Following the same trend observed in the DOE, as concentration increases, particle size increases. The role of particle size is important when it comes to creating mineral bridges between fibers, which can lead to adhesion between fibers to facilitate better stress distribution between them. The length scales in inter‐ and intra‐yarn regions are also important when it comes to bridge formation. Essentially, the diameter of flax fiber is usually between 10 and 20 µm. The intra‐fiber spacing is usually as small as 10 µm or less, whereas it is possible to form particles within a range of sizes between 1 and 50 µm. Due to this inherent variability in the length scales of fibers, intra‐fiber gap, and particle size, the formation of bridges is determined by a combination of the fiber diameter and intra‐fiber gap.

**FIGURE 3:**
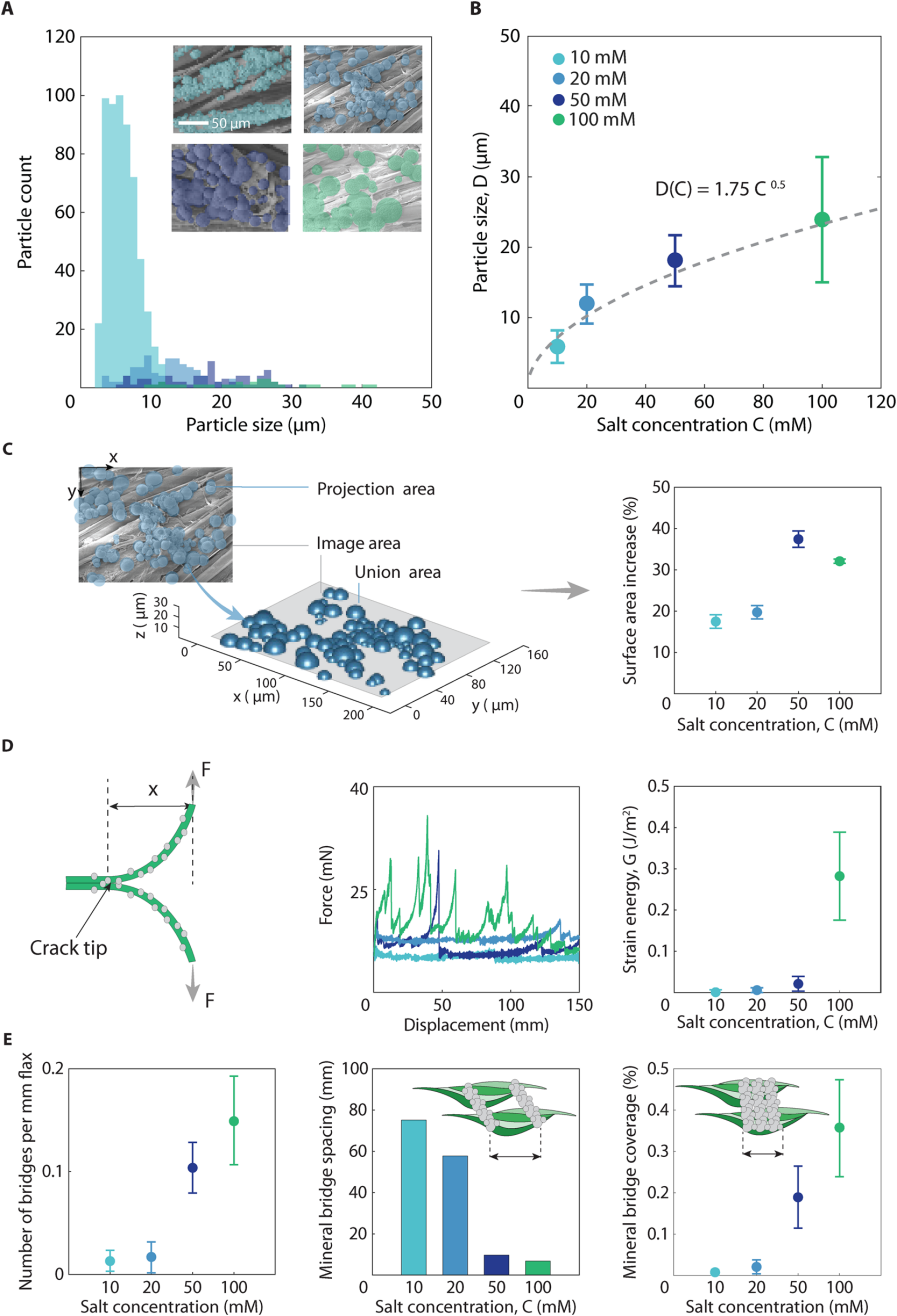
Formation of BM particles on the flax fiber and their effect on dry fiber properties. (a) Particle count versus particle size bar plot to show the distribution prepared by image processing the SEM images given for a wider range of salt concentrations. (b) As the salt concentration increases, particle count reduces and the particle size increases. (c) SEM image of 20 mM sample indicating the particles in color and the increase in surface area obtained for the range of salt concentrations. (d) Representative force displacement curves of peel test of flax yarns to determine the bridging effect of the dolomite‐like particles on the flax fibers. (e) The number of bridges per mm of flax and average bridge spacing along the yarns, and estimated mineral bridge coverage.

SEM images of a variety of fibers treated with different salt concentrations, at 30°C of stationary incubation, are shown in Figure 3a. For a given salt concentration, the different particle sizes and how often they occurred on the fiber surfaces were obtained by image processing the SEM images. The bar plot shows that the particle count significantly reduces by around 90% from 10 to 100 mM, whereas the particle diameter increases with an increased salt concentration, about 5 to 10 times from 10 mM to 100 mM. Particle size is dispersed along a wider range with an increasing salt concentration, indicating that even though the particle size increases, they also have up to around 15 times more deviation due to the greater heterogeneity in the material. At 10 and 20 mM salt concentrations, the particles are highly clustered in large amounts, with an overall particle count of up to 600 particles. However, no clear bridging is observed between the fibers through the minerals, whereas in 50 and 100 mM concentrations of treatment, even with 20% to 80% fewer particles compared to 20 mM, fibers were successfully bridged through e particles approximately twice as large with a mean diameter of 18 to 23 µm. Figure 3b shows the increase in particle size with increasing salt concentration.

An increase in salt concentration results in an increase in particle size and a reduction in particle count. However, increased salt concentration also yields more heterogeneity in particle size, making the deposition more stochastic in size, as evidenced by the standard deviation. The deviation in the particle size increasing as the salt concentration increases suggests that the existing large particles growth is slower than the nucleation of new particles on the fiber surface. Studies on BM and colloidal systems have shown that particle size often follows power‐law relationships with salt concentration due to salt‐induced modulation of supersaturation, nucleation rate, and ionic activity [51, 52, 53]. In our case, the particle size data fit well to a power‐law of the form D(C) = AC^q^, which is consistent with trends observed in dolomite‐like precipitation systems where supersaturation is modulated by salt concentration. The results show that it is possible to create a wide range of particles with reasonably low deviation.

For all salt concentrations used, there is an increase in surface area of the fiber due to the formation of mineral features, as shown in Figure 3c. An example of the methodology for surface area characterization is shown with all images in Figure S4. In the SEM image of the 20 mM sample, the particles were captured and highlighted in blue, and the 3D surface plot of the particles was generated assuming hemispherical particle formation. The surface area increase was calculated by $\frac{Area-Projectionarea}{Imagearea}$. The surface area increase corresponding to each salt concentration was plotted on the right. This means that the surface of the fibers can be roughened through BM processes. For 10–50 mM salt concentrations, the increase in surface area tends to increase together with the corresponding particle size. At 100 mM concentration treatment, there appears to be a threshold in the surface area increase. Even though the size of the particles was larger for the 100 mM samples, their surface area was much smaller. The larger quantity of particles in the 50 mM samples results in a higher surface area. This indicates that for maximizing the surface area, there is a threshold in the BM process. The surface area increase is consistent, considering the standard deviations obtained for each salt concentration, and the surface area of the fibers can be increased through biomineralization. In the 10 mM samples, the surface area increase was around 18%. This value slightly increased to 20% for the 20 mM samples and peaked at almost 40% in 50 mM. As the treatment concentration increased to 100 mM, a drop of almost 6% was observed. A consistency in surface area for a given salt concentration was observed, as its variation was not more than 1%. The surface area increases for different salt concentrations used were highlighted, supporting our hypothesis that the surface of the flax fibers roughens through the deposition of mineral particles.

Bridging between yarns was achieved through dolomite‐like deposition, as evidenced by the strong physical attachment observed after treatment, as well as the SEM images (Figure S8). Following BM, adjacent yarns exhibited cohesive bonding that required the application of mechanical force for separation, whereas untreated yarns remained completely independent and showed no adhesion. This effect was further studied by measuring the peeling force between two bridged yarns (Figure S7), as one exemplary force‐displacement curve from each concentration is provided in Figure 3d. The force‐displacement curves were recorded for four samples per concentration (Figure S9), showing that as the salt concentration of the treatment increased, the force required to pull the yarns apart increased gradually. On the left, the illustration of the double cantilever beam test on flax yarns is shown, where minerals are fractured as the crack tip progresses, with x representing the distance from the loading axis to the crack tip and *F* representing the applied force. In the middle, this behavior is recorded and force displacement curves were plotted for a single sample from each treatment concentration, where the peaks correspond to the force required to break the bridges. Strain energy is calculated and plotted for each treatment concentration at the right using the first three maximum peaks per concentration, which is followed by a minimum 5x drop in force amplitude with less than ‐0.01 N/mm gradient. The presence of large spikes in the force indicates strong bridging between the yarns. An examination of the force–displacement curves reveals that the major peaks for the 100 mM sample reached approximately 0.032 N, which is nearly an order of magnitude higher than those recorded for the 10 mM sample (0.002 N). This behavior was translated into strain energy calculated using the double cantilever beam theory:

$$G=\frac{96F^{2}x^{2}}{E_{yarn}\pi d_{yarn}^{4}}$$

where *F* is the applied force, *x* is the crack length, *E_yarn_* is the yarn modulus, and *d_yarn_* is the yarn diameter. Here, *E_yarn_* was calculated through a cantilever beam test (Figures S5 and S6). For the 10 mM sample, *G* was approximately 0.0036 J/m^2^, increasing to around 0.0238 J/m^2^ at 50 mM and reaching 0.2847 J/m^2^ at 100 mM, rising to more than 10 times that of 50 mM. Although the peak force for 50 and 100 mM samples was similar at certain locations, the strain energy was significantly higher for 100 mM. Higher treatment concentrations produce more cohesive bridging and lead to a tougher material. The high standard deviation for the 100 mM sample is a result of the variability in the yarn stiffness and in the deposition and the material itself.

The number of load peaks in the force‐displacement curves also increased systematically with concentration, indicating intensified bridging between the yarns. More frequent bridging, together with the average size of the particles, resulted in an increased mineral bridge coverage with increased BM levels. This behavior was captured and plotted in Figure 3e. On the left, each peak was assumed to correspond to the separation of a single bridge. The resulting number of bridges is divided by the length of the test sample and reported per mm length along the fiber. The average bridge spacing along fiber length for each sample was calculated by dividing the length of the sample, 150 mm, by the number of bridges. By multiplying the number of bridges by the average size of the particles for each concentration, the mineral bridge coverage was calculated. For these calculations, we assume that each bridge is formed by a single particle area, and bridges are distributed homogeneously over the length of the samples.

The number of bridges per mm increased by an order of magnitude from 0.013 for 10 mM to around 0.15 for the 100 mM sample, which means that the yarns become more interconnected. This is likely to increase the rigidity and structural integrity of the entire yarn. The increased number of bridges had a direct influence on the average bridge spacing, with 80 mm spacing for 10 mM reduced almost 90%, below 10 mm for 100 mM. Furthermore, mineral bridge coverage increased almost 40‐fold, reaching 0.4% for 100 mM in comparison to 10 mM samples. This suggests that the material is transitioning from a loosely connected state to a network structure, as is more common in tough biological materials such as nacre.

Nacre provides a well‐established benchmark for evaluating platelet–platelet connectivity via mineral bridges, which play a crucial role in defining interfacial load transfer and overall toughness. Its characteristic mineral bridge diameters lie in the 10–50 nm range, forming a highly optimized nanoscale network that enhances their toughness and strength. When interpreting these natural length scales, the mineral bridging fraction can be defined as the ratio of mineral bridge area and platelet area. The resulting values of 0.05–0.2 for nacre [54] reflect a relatively dense and mechanically efficient interplatelet coupling network. For our composites, this ratio was estimated as the projection area of a particle, multiplied by the number of peaks per mm determined from Figure 3d, distributed over the surface area of a single flax fiber with a diameter of 30 µm. The mineral bridging fraction was calculated as 0.006 for 100 mM, which indicates a mineral bridge system that is an order of magnitude more sparsely connected than in biological nacre. Even with a smaller fraction of bridges, our results show that the biomineralization strategy can create mineral bridge connectivity that enhances the mechanical properties of the material.

These insights help to understand how different treatment concentrations affect the intra‐yarn bridging, indicating improved bridging and the formation of a denser material network as the salt concentration increases. Next, we investigate the effect of BM precipitation on the mechanical performance of biomineralized flax fiber‐reinforced polymer composites in Figure 4.

**FIGURE 4:**
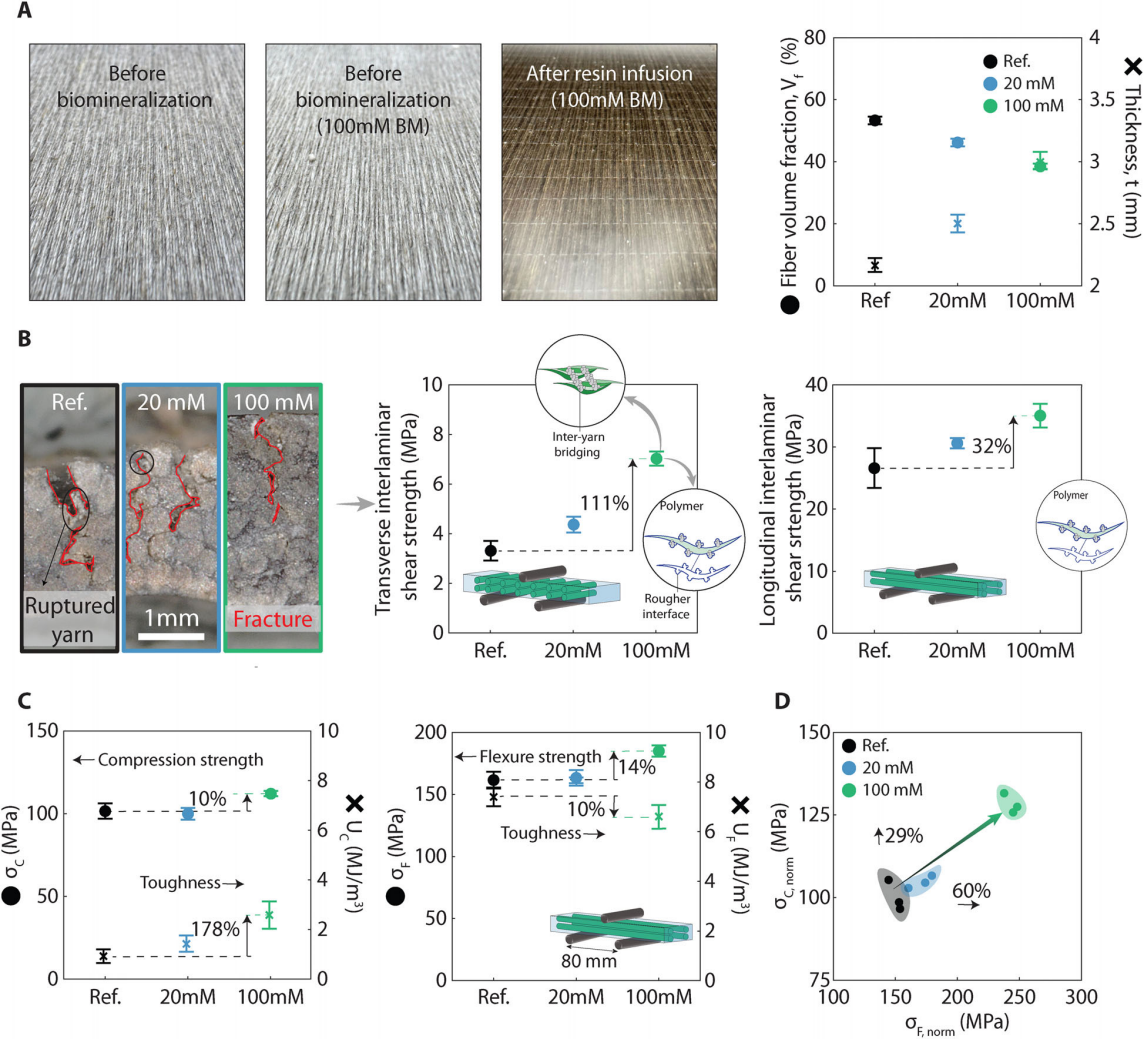
Biomineralization of the flax fibers enhances the compressive strength, interfacial shear strength, and flexural strength of their composites. (a) Photos of flax fabric before and after BM, infused samples, and the corresponding thickness and fiber volume fractions of the samples prepared with different treatment concentrations. Ref. indicates reference samples without BM. (b) Short beam shear setup, microscopy images of transverse samples (l‐r: Ref., 20 mM and 100 mM BM) highlighting the fracture pattern in red lines and the ruptured yarn in black circles. The interlaminar shear strength increases both in the transverse and longitudinal directions. (c) Compressive and flexural tests. *σ*_C_ is the compressive strength, U_C_ is the compressive toughness, *σ*_F_ is the flexural strength, and U_F_ is the flexural toughness. An increase in compressive strength, flexural strength, and compressive toughness followed by a reduction in flexural toughness. (d) Ashby plot of normalized flexural and compressive strength comparing different flax composites and BM flax composites.

Larger particles can form a more rigid material network, creating intensified bridges between the yarns in a fabric. On the other hand, due to the increased particle size and number of particles, the interlaminar gap between layers of fabric becomes larger, resulting in poor compressibility. This effect was captured and plotted in Figure 4a. Photos of the flax fabrics before and after 100 mM BM, together with the vacuum‐infused 100 mM BM flax composite sample, can be seen from left to right.

The *V*_f_ drops from 53% to 38% for the 20 and 100 mM samples, respectively. As the volume of the deposited particles increased with salt concentration, it provided more free volume between each layer of fabric. Reducing *V*_f_ should correspond to lower fiber‐dependent mechanical performance; however, we found improvements in the interlaminar shear strength (ILSS), compressive, flexural, and toughness performance.

This behavior was recorded by a short beam shear test in both transverse and longitudinal fiber arrangements as shown in Figure 4b. On the left, the transverse test setup can be seen, where the fracture is analyzed, and the crack path is highlighted on the samples. The transverse interlaminar shear strength, with the major contributing mechanisms for the improvement, is shown in the middle. The interlaminar shear strength of the longitudinal samples, on the right, shows an improvement similar to the surface area increase in percentage.

In the short beam shear test on transverse samples, the untreated sample exhibited catastrophic failure. The crack not only propagated through the periphery of the yarns but also caused the yarns to rupture. This indicates that the fibers in a yarn were weaker compared to those in the BM samples. The bridging and increasing particle size at higher concentrations resulted in reduced crack growth, as evidenced by the reduced distance between the two adjacent surfaces of the fracture. No clear yarn rupture was observed in the 100 mM sample, which can be the result of the particle bridging between the individual fibers of a yarn. This was translated into the transverse ILSS as the 100 mM sample achieved more than double the ILSS of the Ref. samples, reaching up to 7 MPa. This increase suggests that the particles on the flax fibers improve the interface between the fibers and the polymer by introducing a rougher surface and forming inter‐yarn bridges. It is important to note that short beam shear stress is not a direct measure of the interfacial adhesion. We partially observe the effect of improved mechanical interlocking, particle bridging forming cohesive interlinks between the yarns, and changes in the composite microstructure. The rougher surface of the fibers eventually improves the mechanical interlocking between the fibers and the polymer. A part of this increase can be attributed to the roughening of the fibers, as the surface area of the yarns can be increased up to around 32% for the 100 mM, as previously identified. The remaining contribution can be attributed to mineral bridges between neighboring yarns, which enhance inter‐yarn mechanical coupling and increase resistance to transverse shear failure. The ILSS of the longitudinal samples increased from around 26 to 34 MPa, an increase of 32%, which is almost the same as the increase in surface area of the fiber for 100 mM samples, suggesting that ILSS of the composites in the longitudinal direction might be dominated by the roughening of the fiber surface through BM.

Compressive and flexural characterization of the samples can be seen in Figure 4c. On the left, compressive strength and toughness of the samples are plotted. The compressive strength and especially compressive toughness show a remarkable increase through BM despite the addition of a particle volume fraction of only 1.7%. The compressive strength of the fibers was increased by 10% from around 101 MPa for Ref. samples to 112 MPa at 100 mM concentration. Furthermore, the samples were toughened by the BM treatment, in which, for the 100 mM sample, it is almost three times the toughness of the Ref samples, reaching up to 2.6 MJ/m^3^. Unidirectional composites tend to fail by fiber buckling and kinking under longitudinal compression, which is sensitive to interfacial sliding and matrix shear stiffness. A stronger interface resists interfacial shear and better constrains waviness, and can alter the instability mode from localized kinking to a more distributed deformation response involving coupled kinking and buckling [55, 56], as evidenced by the fracture images of the compressive samples (Figure S12). This transition reflects a more effective load transfer and stronger mechanical interaction between the fiber and matrix. As a result, the composite can sustain higher stresses and exhibit increased energy absorption capacity.

Similarly, the flexural strength of the composite increased by 14% from around 161 to 185 MPa for 100 mM concentration, while the flexural toughness decreased by 10% from 7.4 to 6.6 MJ/m^3^. This behavior can be attributed to improved interfacial adhesion and bridging mechanisms introduced by the BM. In bending, flexural strength is governed by the outer fibers onthe tensile side. Improved interfacial bonding enables more efficient load transfer to the fibers, resulting in higher strength. This is further supported by the pronounced tensile‐side delamination in the treated samples (Figure S12), indicating that sufficient interfacial integrity is achieved to develop interlaminar stresses. In contrast, the untreated composites exhibited more diffuse deformation, suggesting limited stress transfer and interfacial sliding. However, flexural toughness is largely governed by energy‐dissipating mechanisms such as fiber pull‐out, interfacial debonding, and frictional sliding during crack propagation. Strengthening the interface suppresses these mechanisms by restricting interfacial slip.

Compared to the unidirectional untreated natural fiber composites, BM has the potential to offer improved performance, although the magnitude of the benefit is sensitive to processing‐induced changes in laminate architecture. The performance of BM flax/epoxy composites created in this work is shown in an Ashby plot in Figure 4d. Compressive strength andflexural strength data were plotted for reference flax/epoxy, as well as the BM flax/epoxy composites, which were normalized to 50% V_f_ to facilitate an indicative comparison across different fiber contents. The compressive failure is governed by a shear instability where the critical stress scales with the square root of longitudinal stiffness and the in‐plane shear modulus of the laminate [57]. Because the kink‐band initiation occurs mainly in the matrix rather than the fibers, the shear modulus is known to be matrix‐dominated and only weakly affected by the changes in V_f_. The ratio of compressive strength at two different V_f_ leaves a normalization that only depends on the measurable longitudinal stiffness $\sigma_{V_{f}}=\sigma_{V_{f}0}\sqrt{\frac{E_{V_{f}}}{E_{V_{f}0}}}$, where $\sigma_{V_{f}}$ is the compressive strength at target V_f_, $\sigma_{V_{f}0}$ is the measured compressive strength at measured V_f_, $E_{V_{f}}$ is the modulus calculated for the target V_f_ by the stiffness normalization and $E_{V_{f}0}$ is the measured stiffness at the measured V_f_. On the other hand, flexural strength was normalized using a linear scaling with V_f,_ based on the principle that bending failure is governed by tensile fracture of the outer fibers, making the load‐bearing capacity directly proportional to the amount of reinforcement present [58]. After normalization, BM samples exhibited around a 29% increase in compressive strength and a 60% increase in flexural strength compared to reference epoxy/flax samples. When benchmarked against literature data for flax‐based composites, the BM flax/epoxy composites show a favorable combination of specific compressive strength and stiffness after normalization to 50% fiber volume fraction, performing comparably to, and in some cases exceeding, the reported literature values (Figure S13). The results highlight the promising potential of BM in reinforcing natural fibers, improving the load transfer and structural integrity of the resulting composites. However, the normalized values are best interpreted as an indicative comparison (non‐normalized data in Figure S11), as biomineralization can modify fiber packing and laminate architecture through changes in porosity, resin‐rich regions, and yarn compaction.

While prior studies of nacre‐like materials have emphasized the central role of mineral bridge density in governing shear strength and interfacial connectivity, the 111% increase in ILSS observed for the BM flax/epoxy composites suggests a substantial improvement in interlaminar load transfer, which may be associated with BM‐induced connectivity. However, the ILSS is also influenced by fiber volume fraction, fiber architecture, compaction, void content, and processing route. Within the limited available literature on treatment‐induced ILSS improvements in flax/epoxy composites, plasma‐treated and alkaline‐treated flax/epoxy laminates have been reported to show improvements of approximately 40% and 27%, respectively [59, 60]. Considering both studies used woven fabric architectures and that their processing parameters differ, these reported values are not directly comparable to our work. Nevertheless, they provide a useful qualitative benchmark for contextualizing the ILSS enhancement achieved through BM, which produces bridge features between the fibers. Despite exhibiting an order of magnitude lower bridge density than nacre, the mechanical performance of our natural fiber composite system remains robust, indicating that connectivity alone does not fully dictate load transfer efficiency in fiber‐reinforced polymeric materials. Instead, the hierarchical flax fiber architecture, which spans from microfibrillar organization to lumen‐scale features, provides alternative pathways for stress redistribution, including fibrillar sliding, interfacial friction, and progressive debonding.

The lower connectivity compared to nacre may promote enhanced energy dissipation through extrinsic mechanisms such as fiber pull‐out and crack deflection, thereby mitigating the brittleness often associated with highly mineralized systems. Overall, these findings point toward a regime in which mechanical performance arises from a synergy between sparse but efficient mineral bridging, the intrinsic structural complexity of the cellulose microstructure within flax fibers, and the reinforcing polymer matrix.

This has two key consequences: first, the enhanced interface suppresses interfacial slip, enabling more effective load transfer into the fiber phase, and second, the mineral bridges provide lateral support to the fibers, increasing their resistance to microbuckling by constraining transverse deformation. The combined effect of increasing the composite shear performance shifts the governing failure mechanism from early interfacial decohesion toward more distributed, higher‐stress deformation, which manifests macroscopically as a ∼30% increase in compressive strength and the high toughness values that we measured. As a result of these combined effects, biomineralization has the potential to increase the specific compression strength of flax by 40% to 113 MPa cm^3^/g, closing the gap with glass fiber composites, where the typical specific compression strength is 175–300 MPa cm^3^/g if the fiber volume fraction can be preserved after the treatment.

To address the environmental and scaling trade‐offs of the biomineralization method, a preliminary screen‐level inventory analysis was conducted (Table S1). The analysis revealed that the overall footprint is predominantly driven by energy consumption during laboratory processing rather than by the raw materials used, highlighting that the approach has potential as a sustainable modification to flax fiber composites.

## 3 Conclusions

Imaging and testing of BM flax yarns revealed that the key mechanisms driving the improvement in compressive, interfacial, and flexural behavior of the resulting BM flax/epoxy composites are the increased surface area and bridging via nucleated particles that create solid networks within the fabric structure. As a result of BM, normalized compressive strength, normalized flexural strength, and ILSS of the composites improved by 30%, 111%, and 60%, respectively. The compressive samples exhibited an almost three times higher toughness through the amplification of extrinsic toughening effects mediated by the biomineral precipitations within the composite material.

Future research should prioritize optimizing mineralization kinetics and particle distribution to maximize homogeneity and mechanical gains while maintaining high fiber volume fractions. Biomineralization may be implemented at distinct stages of the manufacturing process to promote homogeneous surface roughening alongside spatially localized mineral deposition, for inter‐yarn bridge formation and improved control over fiber volume fraction. As scalability remains a key challenge for industrial adoption, large‐scale implementation will require precise control of bacterial activity, nutrient cycles, and dolomite‐like precipitation to ensure uniformity whilst maintaining high volume fractions of flax‐fiber within the composite.

Environmental and economic performance assessments represent important next steps, where our preliminary evidence is highly promising. In addition, considering the lignocellulosic structure of flax fibers, long‐term effects of biomineralization on moisture uptake, ageing behavior and environmental durability of the resulting composites must be carefully studied. Finally, coupling BM with advanced composite manufacturing techniques such as additive manufacturing offers a pathway to consistent, high‐performance CO_2_‐capturing materials in sustainable structures.

## 4 Experimental Section

### 4.1 Bacterial Stock Preparation

*S. pasteurii* DSM33 was grown in 100 mL of SP2 medium at 30°C and 180 rpm for 48 h. The culture was centrifuged in two balanced 50 mL tubes for 5 min at 3,220 × *g*. The supernatant was carefully decanted, and the remaining pellets were resuspended in 5 mL of fresh SP2 medium and mixed with an equal volume of % 80 (v/v) sterile glycerol solution, resulting in ≈15 of OD_600_. The suspension was aliquoted into a 2 mL cryotube, and stored at −80°C. SP2 medium used in the stock preparation contains 20 g L^−1^ yeast extract, 10 g L^−1^ ammonium chloride, 10 µM nickel (II) chloride, with the pH adjusted to 8.5 using sodium hydroxide pellets.

### 4.2 Biomineralization (BM) treatment

Flax yarns were extracted from BCOMP Amplitex UD 280; the same fabric was used to prepare the composite samples. To biomineralize the flax, 10 g of yarns were immersed in 1 L of BM solution containing different total salt concentrations (10 to 100 mM), with the urea concentration fixed at three times the total salt concentration. In addition to these precursors, the solution contained 10 g L^−1^ peptone, 5 g L^−1^ yeast extract, 10 g L^−1^ ammonium chloride, and was inoculated with 1 mL of *S. pasteurii* glycerol stock solution. The BM solution, together with flax yarns, was incubated at 30°C and 125 rpm for 24 h. To terminate the reaction and deactivate the bacteria, the solution was boiled for 10 min, and after that, the flax yarns were removed, washed, and dried in a Thermo Scientific VT6060P vacuum oven for 3 h at 70°C. Fabrics used in the composite samples were biomineralized using the same protocol. However, the fabrics were clamped from the ends to prevent destroying the fiber orientation during BM. This was done using a clamp designed in‐house and printed using a polycarbonate filament in a Prusa MK3S 3D printer (Figure S10).

### 4.3 Vacuum Infusion

Epikure/Epikote 04908 epoxy system was degassed for 40 min after mixing. Infusion was made at 50 mbar; after the resin reached the exit, pressure was increased to 500 mbar, and the part was cured at room temperature.

### 4.4 Mechanical Testing

The compression (140 × 13 × 3 mm samples), short beam shear (15 × 5 × 3 mm samples) and flexural tests (100 × 12 × 3 mm samples) were made in Zwick 20 kN Universal Test Machine (ZwickRoell, Germany) following ASTM D6641‐09, ASTM D2344M‐22 and ASTM‐D7264‐D7264M‐21, respectively. Microscopy and SEM images were taken by Keyence VR 5000 digital microscope and JEOL JSM‐7500F, respectively.

## Author Contributions

**Conceptualization**: D.S., K.M., and J.T. **Data curation, formal analysis**: D.S., I.N., and J.J.O. **Funding**: K.M. and J.T. **Investigation**: All. **Methodology**: D.S., I.N., J.J.O., and M. E.‐A.T. **Supervision**: K.M. and J.T. **Validation**: D.S. and K.M. **Visualization**: D.S., K.M., J.T., and I.N. **Writing – original draft**: D.S., K.M., and J.T. **Writing – review, editing**: All.

## Conflicts of Interest

The authors declare no conflicts of interest.

## Supporting information




**Supporting File**: adma74543‐sup‐0001‐SuppMat.docx.

## Data Availability

The data that support the findings of this study are available from the corresponding author upon reasonable request. Source codes will be made available through our GitLab page.
