# Supplementary material for: Flax Composites With Improved Interfacial Strength Through Microbially Induced Mineral Precipitation
Source: Adv Mater. 2026 Aug 8;38(56):e74543. doi: 10.1002/adma.74543 (PMC13648884; doi:10.1002/adma.74543)
Supplement: Supplementary file 1 [file ADMA-38-e74543-s001.docx]

**SUPPORTING INFORMATION:**

**Flax Composites with Improved Interfacial Strength through Microbially Induced Mineral Precipitation**

Deniz Sayinbas^1^, Ingo Nettersheim^1^, Jeong-Joo Oh^3^, Marie-Eve Aubin-Tam^3^, Julie Teuwen^2^, Kunal Masania^1*^

^1^Shaping Matter Lab, Faculty of Aerospace Engineering, Delft University of Technology, Kluyverweg 1, 2629 HS Delft, Netherlands

^2^Aerospace Structures and Materials, Faculty of Aerospace Engineering, Delft University of Technology, Kluyverweg 1, 2629 HS Delft, Netherlands

^3^Bionanoscience Department, Faculty of Applied Sciences, Delft University of Technology, Van der Maasweg 9, 2629 HZ Delft, Netherlands

*Corresponding author: [k.masania@tudelft.nl](mailto:k.masania@tudelft.nl)

**Contents:**

Image processing of DOE images

Image processing for particle size and surface area increase

Characterization of the yarn stiffness

Double cantilever beam test

Biomineralization of flax fabrics

Discussion on sustainability and life cycle assessment (LCA) of biomineralized flax composites

**Image processing of DOE images**

**Figure S1.** The figure displays the evolution of the SEM images during image processing: Particles were first isolated adding a black background to the area where no particles are present. Later particles were detected by ImageJ, adjusting the threshold and converting the image into binary form. The parameters used in the biomineralization of the corresponding yarns in the images given on the left. Image processing sequence followed is given from left to right.

**Figure S2.** The SEM image is processed to capture the location of the particles and the size of the particles, assuming that the particles are perfect circles. The image area is discretized into quadrats and the number of particles centered in each quadrat was counted. The variance-to-mean ratio is then calculated by dividing the variance of the number of particles in each quadrat by the mean of the number of particles in each quadrat. This gives a rough estimation of particle randomness, homogeneity and clustering.

**Figure S3: Repeated measurement for the dominant screening trend: salt concentration. a** The effect of salt concentration on particle size. **b** Effect of salt concentration on surface coverage. **c** Effect of salt concentration on VMR. The high standard deviation for 10mM is due to the highly clustered deposition at varying intensities in such small particles.

**Discussion on observed effects in the DOE**

Salt concentration significantly affects the size and surface coverage of the flax. For 100mM concentration, particles are as large as twice the size of 20mM samples. This has also been captured by the p-value, which is as low as 0.036, showing the strong effect of salt concentration to create particles in different size. This is important since it allows tuning the microstructure due to the range of particle size that could be consistently created on the flax surface, which could potentially lead to different functional microstructure-governed mechanisms that can influence the mechanical behavior of the material. Moreover, surface coverage of the particles can also be significantly influenced by the salt concentration, as evidenced by the p-value of 0.038. Essentially, larger particles obtained by the higher salt concentration and availability of more salts in the solution lead to a better coverage, although the deposition yield reduces.

Temperature has an influence on the rate and morphology of mineral deposition. Literature reports that lower temperatures (approximately 20 °C) promote slower precipitation kinetics but yield more uniform and well-defined crystal structures, whereas elevated temperatures (around 50 °C) accelerate initial nucleation and result in irregular particle sizes and reduced overall deposition [1], [2]. In this study, a narrower temperature range was selected based on both prior findings and preliminary experimental observations. Notably, the DOE indicates that while particle size variability remains minimal within the optimized range, temperature continues to exert an effect on deposition yield and surface coverage, even at 27 °C and 30 °C as evidenced by the p-value being 0.107 and 0.078 being either close to 0.1 or smaller than 0.1 for deposition yield and coverage, respectively. These effects are explained by the relationship between the metabolic activity of the bacteria and pH [3]. Within the range of 27–30 °C, temperature plays a critical role in enhancing bacterial metabolism and urease activity, thereby accelerating urea hydrolysis and promoting a rapid increase in pH levels. The higher pH of the solution reduces the solubility of Ca2+ and shifts the carbonate equilibrium towards ${CO}_{3}^{2\text{-}}$ , driving precipitation. This optimal enzymatic performance ensures efficient carbonate availability and high deposition rates, thus surface coverage. Nevertheless, even minor temperature variations within this narrow range can alter reaction kinetics and pH dynamics sufficiently to influence precipitation efficiency, underscoring the inherent sensitivity of the processes to thermal conditions.

Stirring can influence the process by prohibiting the growth of the particles, resulting in smaller particles on the fibres and can also affect the deposition rate. The p-value being as small as 0.008 shows that the effect of stirring is more dominant on particle size. We believe the movement in the liquid also has a strong effect on the biological processing. More shear in the liquid counteracts the electrostatic attraction between the negatively charged fibre surface and positively charged magnesium and calcium ions, preventing particles from nucleating on the surface of the fibres. Conversely, mixing, facilitates better dispersion, and breaks local chemical gradients that form, to more effectively use up nutrients and ions in the solution. Of the two effects, we found that not stirring in our case, leads to larger deposition yield, and more homogenous precipitation.

I**mage processing for particle size and surface area increase**

**Figure S4.** Characterization of surface area increase for the whole range of BM concentrations 10, 20, 50 and 100 mM, color coded. The inflated particles as hemispheres were formed in MATLAB using the size and particle coordinates as in **Figure S2.**

**Characterization of the yarn stiffness**

**Figure S5.** Images taken in the cantilever beam test. Yarns with a length of 5 cm were clamped from one end and a weight of 50 mg was attached to the free end. The deflection of the yarns was measured to calculate the stiffness.

**Figure S6.** The illustration of the cantilever beam test is shown on the left, where W is the weight of the yarn, F is the force applied by the external weight and $\delta$ is the deflection of the yarn. The weight of the particles and then the total volume of the particles were calculated by the change in weight of the yarns after BM. Assuming that the total volume of the particles forms a homogenised hollow cylinder shell around the yarn, the diameter of the yarn is assumed to be the sum of the non-biomineralized yarn diameter and the thickness of the hollow cylinder shell, which is shown on top of the setup illustration. Corresponding yarn diameter and yarn weight were plotted in the middle. Yarn stiffness was calculated and plotted for each concentration of BM at the right.

The apparent modulus $E$calculated from the cantilever beam relation

$$E_{yarn}\text{=}\frac{\frac{FL^{3}}{3}\text{+}\frac{WL^{4}}{8}}{I\delta}$$

is highly sensitive to geometric scaling because $I$, the second moment of area for a circular cross-section, increases with the fourth power of the diameter ($I\text{=}\pi d^{4}\text{/}64$).

**Double cantilever beam test**

**Figure S7.** Double cantilever beam test setup indicating the loading axis and crack tip. Time in a test advances from left to right.

**Figure S8.** SEM images showing the mineral bridges on 100 mM samples. SEM images shows that the mineral bridges can for in different ways. On the left the mineral bridge is formed by cluster of particles connecting the yarns. On the right, a single fibre from yarn 2 approaches towards yarn 1, forming a mineral bridge through the length of the fibre. For the ease of the calculation regarding the connectivity factors like, mineral bridge spacing and mineral bridge coverage, bridges are assumed to be formed by a single particle in the main manuscript.

**Figure S9.** Force-displacement curves for each sample are shown at the left. The data was smoothed and plotted at the right, with the peaks at the force captured without any noise. The number of peaks in each data set corresponds to the number of bridges within the given sample. The yarn length used is 150mm. A 300 mm displacement was set for the test to make sure two yarns are completely separated at the end of the test.

**Biomineralization of flax fabrics**

**Figure S10.** To keep the fabrics in place without any deformation, a clamp was designed and 3D printed from polycarbonate. The design of the clamp, indicating the function of the parts, is shown at the left. The clamped fabrics are positioned in the middle, and the setup used for biomineralization of the fabrics is shown on the right.

**Mechanical characterization of BM flax composites**

**Figure S11.** **a** Compressive test setup and failed samples with different BM concentrations. **b** The strain-stress curves of compressive test samples. **c** Stiffness dropped around 30% for the 100mM compared to reference samples with no BM, which is mainly due to the reduction of the V_f_ upon BM. When the data is normalized to 50% V_f_, the stiffness of the 100 mM sample was almost the same as that of the reference sample. **d** The back-calculated stiffness of the fibres from the compression test remained almost unchanged for low concentration. However, it decreased in high concentrations. **e** The strain-stress curve for the 3-point bending test. **f** Reduction in fibre volume fraction led to a 20% decrease in flexural stiffness from around 11 GPa to around 9 GPa. When the data is normalized to 50% V_f_, 25% increase was observed for the 100mM sample compared to the reference samples. **g** The force-deflection curves for the short beam shear test in longitudinal samples. **h** The force-deflection curves for the short beam shear test in transverse samples.

**Figure S12.** **a** Compression test, failed samples. Nontreated, 20mM,100mM samples from left to right respectively. Clear kinking observed in the compression nontreated sample, whereas kinking was combined with the buckling in BM samples. **b** 3 point bending test, failed samples. Nontreated, 20mM,100mM samples from left to right respectively. Delamination at the tension side was observed in the BM samples whereas, no clear delamination is present in the non-treated samples.

**Figure S13.** Ashby plot of specific compressive strength and specific compressive stiffness for UD flax composites reported in the literature and untreated 20mM BM and 100mM BM flax yarn composites from the present work. Literature data includes two flax/epoxy systems where UD flax yarns are used (black squares) and three UD flax /epoxy systems where aligned UD fibres are used (not flax yarns) (black x), with the corresponding references indicated next to each data point. It is important to note that the unidirectional flax tape architecture used in the data indicated in black x is different from the flax yarns used in this work and the literature data indicated in black squares. To account for differences in fibre volume fraction, all data were normalised to a fibre volume fraction of 50% using the same normalisation methodologies stated in the main manuscript for compressive strength and compressive stiffness. Standard deviations for each literature data point are included as available.

**Discussion on sustainability and life cycle assessment (LCA) of biomineralized flax composites**

A preliminary carbon-footprint estimate was carried out for a five-layer laminate by summing the CO_2_ equivalents for the principal biomineralisation reagents, flax fabric, epoxy, and the electricity required for drying, incubation, boiling, and vacuum-assisted processing. Using the Dutch electricity as the basis for calculation (of 0.483 kg CO₂-eq /kWh), the estimated footprint was 3.9 kg CO₂-eq per laminate, which is lower than the 5.8 kg CO₂-eq reported for an alkaline-treated flax/HDPE laminate of similar volume [9]. All the calculations, assumptions, amount of electricity consumption for the devices used, ingredients included in the calculation and sources are stated in Table 1. The resulting total carbon footprint of 3.9 kg CO₂-eq per laminate is calculated for a laminate size of 180mm x 110 mm x 3 mm, whereas in the reference value 5.8 kg CO₂-eq, the laminate size used was 160mm x 160 mm x 2 mm. It must be acknowledged that the high-density polyethene (HDPE) was used in the reference paper, whereas in this work of biomineralized flax composites, epoxy resin was used. The results indicate that the overall carbon footprint is predominantly driven by electricity consumption. These findings highlight that the key determinant of environmental impact is material processing (3.3 kg CO₂-eq per laminate) than from the raw materials, chemicals and consumables used (0.6 kg CO₂-eq per laminate).

Although this comparison remains approximate and does not represent a full LCA with definitive values, it nevertheless points out that the biomineralisation route is not inherently less favourable than conventional alkaline treatment from a carbon-emissions perspective.

| **Category** | **Inventory item** | **Counted in total?** | **Activity data** | **Equation used** | **EF / power input used** | **Contribution (kg CO₂e)** | **Assumption and how the EF or estimate was reached** | **Main source(s)** | **Data quality** |
| --- | --- | --- | --- | --- | --- | --- | --- | --- | --- |
| Material | Peptone | Yes | 25 g = 0.025 kg | CO2e = 0.025 kg × 5.0 kg CO2e/kg | 5.0 kg CO2e/kg | 0.12500 | Engineering proxy for a protein-rich processed nutrient. No robust public product-specific cradle-to-gate PCF was identified during the search, so a conservative mid-range placeholder was used. This should be replaced first if better data become available. | No direct public PCF identified; assumption-based placeholder. | Low |
| Material | Yeast extract | Yes | 12.5 g = 0.0125 kg | CO2e = 0.0125 kg × 3.32 kg CO2e/kg | 3.32 kg CO2e/kg | 0.04150 | Direct use of a public product carbon footprint expressed per kg of product. Product type (yeast extract powder) and unit matched the required inventory item well, so no conversion other than g→kg was needed. | [10] CarbonCloud “Yeast extract (powder), EU | Medium–High |
| Material | Ammonium chloride | Yes | 25 g = 0.025 kg | CO2e = 0.025 kg × 1.27 kg CO2e/kg | 1.27 kg CO2e/kg | 0.03175 | Direct use of a public product carbon footprint for the exact chemical; only a mass conversion from g to kg was required. | [11] CarbonCloud “Ammonium chloride” . | Medium–High |
| Material | Calcium chloride dihydrate (modelled with CaCl2 proxy) | Yes | 18 g = 0.018 kg | CO2e = 0.018 kg × 1.53 kg CO2e/kg | 1.53 kg CO2e/kg (CaCl2 proxy) | 0.02754 | Used a public carbon footprint for anhydrous CaCl2 as a proxy because a product-specific PCF for CaCl2·2H2O was not identified. This likely slightly overestimates the dihydrate on a per-kg basis because the hydrate contains crystal water. | [12] CarbonCloud “Calcium chloride (CaCl2)” . | Medium |
| Material | Magnesium chloride hexahydrate | Yes | 25 g = 0.025 kg | CO2e = 0.025 kg × 0.8 kg CO2e/kg | 0.8 kg CO2e/kg | 0.02000 | Generic inorganic-salt placeholder. No robust public cradle-to-gate PCF for MgCl2·6H2O was identified during the search, so a low-to-mid proxy was used to avoid leaving the item out. | No direct public PCF identified; assumption-based placeholder. | Low |
| Material | Urea | Yes | 50 g = 0.050 kg | CO2e = 0.050 kg × 1.52 kg CO2e/kg | 1.52 kg CO2e/kg | 0.07600 | Direct use of a third-party verified product carbon footprint for urea from Sluiskil, NL. This was selected because it is geographically credible and already provided in kg CO2e per kg product. | [13] Yara / DNV verified carbon-footprint statement for urea . | High |
| Material | Bcomp ampliTex UD 300 flax fabric | Yes | 25 g = 0.025 kg | CO2e = 0.025 kg × 0.70 kg CO2e/kg | 0.70 kg CO2e/kg | 0.01750 | Used Bcomp’s own comparative ecological-aspects indicator for ampliTex flax. The product match is strong (UD flax fabric, 300 gsm), but the figure is not presented as a full EPD with explicit module boundaries in the snippet reviewed, so it is treated as a manufacturer-based proxy for finished fabric rather than a formal PCR-compliant declaration. | [14] Bcomp ampliTex datasheet / ecological aspects chart . | Medium |
| Material | Epikote–Epikure epoxy system (proxy) | Yes | 25 g = 0.025 kg | CO2e = 0.025 kg × 4.6 kg CO2e/kg | 4.6 kg CO2e/kg | 0.115 | Used a verified EPD-style value for a comparable epoxy resin product because a public EPD/PCF for the exact Epikote–Epikure system was not identified. This is therefore a proxy for generic epoxy resin-system production and should be replaced if an exact grade-specific data is available. | [15] Kočí & Loubal epoxy resin LCA study. | High |
| Material | Water (In-house lab-purified / ultrapure water) | Yes | 2.5 L | CO₂e = 2.5 L × (0.0003–0.0010) kg CO₂e/L = 0.00075–0.00250 kg CO₂e | 0.0006 kg CO₂e/L | 0.0015 | Water emissions estimated via energy–water nexus: energy intensity 0.5–3.5 kWh/m³ converted to kg CO₂e/L using electricity factor | [16] Water-energy intensity background | Medium |
| Electricity | Dutch grey-grid electricity factor | Yes | Applied to all electricity-using steps | CO2e = kWh × 0.483 kg CO2e/kWh | 0.483 kg CO2e/kWh |  | Used the Dutch reporting factor for grey electricity valid from 1 January 2026, including upstream emissions. This was selected because the work is being carried out in the Netherlands. | [17] Milieubarometer / Dutch electricity factor . | High |
| Electricity | Incubator at 30 °C for 24 h | Yes | 0.10 kW × 24 h = 2.40 kWh | CO2e = 2.40 kWh × 0.483 kg CO2e/kWh | 100 W average operating power | 1.15920 | Because the exact incubator model was not provided, a 100 W average operating draw was assumed. This was intended as a conservative small-lab estimate. For context, Thermo Heratherm compact incubators are listed around 45 W maximum and ~14 W actual consumption at 37 °C for one compact model, so this assumption may overestimate the incubator contribution if your unit is compact. | [18] Thermo Heratherm incubator documentation [1] Dutch electricity factor | Medium–Low |
| Electricity | Thermo vacuum oven at 70 °C for 3 h | Yes | 0.60 kW × 3 h = 1.80 kWh | CO2e = 1.80 kWh × 0.483 kg CO2e/kWh | 600 W nominal power (small-unit base case) | 0.86940 | Used 600 W as the base-case power draw because Thermo vacuum-oven documentation shows approximately 600 W for a small 0.7 cu ft unit, while larger units can be much higher (up to ~1600 W). Therefore, this step is modelled as a small-unit scenario rather than a guaranteed exact value. | [19] Thermo Scientific vacuum-oven documentation  1. Dutch electricity factor | Medium |
| Electricity | IKA C-MAG hotplate boiling for 10 min | Yes | 1.02 kW × (10/60) h = 0.17 kWh | CO2e = 0.17 kWh × 0.483 kg CO2e/kWh | 1020 W power input | 0.08211 | Used the IKA C-MAG HS 7 listed power input directly. Because the user specified boiling on an Aldrich/IKA C-MAG heater at maximum temperature for 10 min, this is one of the more concrete activity estimates in the model. | [20] IKA C-MAG HS 7 technical data  [17] Dutch electricity factor | High |
| Electricity | Small lab vacuum pump for 24 h infusion | Yes | 0.10 kW × 24 h = 2.40 kWh | CO2e = 2.40 kWh × 0.483 kg CO2e/kWh | 100 W average operating power | 1.15920 | Because an exact pump model was not given, a 100 W average operating draw was assumed. Vendor examples for small oil-free lab vacuum pumps span roughly 30–100 W for compact units, with larger pumps higher; therefore, the value is plausible but still uncertain. | [21] LabTech oil-free vacuum-pump product information  [17] Dutch electricity factor | Medium–Low |
| Vacuum-infusion consumable | Peel ply (optional estimate) | Optional estimate | Area basis: 1.5 × plate area × 85 g/m² = 2.52 g | CO2e = 0.0025245 kg × 5.0 kg CO2e/kg | 5.0 kg CO2e/kg generic polymer-textile proxy | 0.01262 | Estimated from a typical peel-ply areal weight of 85 g/m² and a practical lay-up factor of 1.5× plate area to allow trimming and overlap. The carbon factor itself is a generic polymer/textile proxy, not a peel-ply-specific EPD. | [22] Typical peel-ply areal-weight listings  Generic polymer/textile proxy assumption. | Low |
| Vacuum-infusion consumable | Infusion mesh / flow medium (optional estimate) | Optional estimate | Area basis: 1.5 × plate area × 160 g/m² = 4.75 g | CO2e = 0.004752 kg × 5.0 kg CO2e/kg | 5.0 kg CO2e/kg generic polymer-textile proxy | 0.02376 | Estimated from a common infusion-mesh areal weight of 160 g/m² and the same 1.5× area factor. No product-specific PCF was available, so the same generic polymer/textile proxy was used for CO2e per kg. | [23] Typical infusion-mesh listing  Generic polymer/textile proxy assumption. | Low |
| Vacuum-infusion consumable | Vacuum bag film (optional estimate) | Optional estimate | Area basis: 2.0 × plate area × 63 g/m² = 2.49 g | CO2e = 0.0024948 kg × 5.0 kg CO2e/kg | 5.0 kg CO2e/kg generic polymer-film proxy | 0.01247 | Estimated from a 2× area allowance for bagging and a representative thin-film areal mass (~63 g/m², consistent with ~55 µm-class vacuum bagging film). The CO2e factor is a generic polymer-film proxy, not a product-specific bag-film EPD. | [24] Typical vacuum-bag-film product listings generic polymer-film proxy assumption. | Low |
| Vacuum-infusion consumable | Release film (optional estimate) | Optional estimate | Area basis: 1.5 × plate area × 25 g/m² = 0.74 g | CO2e = 0.0007425 kg × 5.0 kg CO2e/kg | 5.0 kg CO2e/kg generic polymer-film proxy | 0.00371 | Estimated from a thin perforated release film with a nominal areal mass of 25 g/m² and 1.5× area allowance. The carbon factor is a generic polymer-film proxy. | Thin-film assumption informed by typical release-film use; generic polymer-film proxy assumption. | Low |
| Vacuum-infusion consumable | Sealant tape + spiral + fittings (optional estimate) | Optional estimate | Fixed estimate = 5 g = 0.005 kg | CO2e = 0.005 kg × 5.0 kg CO2e/kg | 5.0 kg CO2e/kg generic polymer/plastic proxy | 0.02500 | Fixed bundle estimate for small-process consumables that are difficult to allocate precisely at sample scale. The mass and carbon factor are both broad engineering assumptions and should be replaced with weighed consumables if better accuracy is required. | Engineering estimate; generic polymer/plastic proxy assumption. | Low |

**Table S1:** Detailed assumptions and calculations used in the indicative LCA approximation

**References**

[1] X. Sun, L. Miao, T. Tong, and C. Wang, ‘Study of the effect of temperature on microbially induced carbonate precipitation’, Acta Geotech., vol. 14, no. 3, pp. 627–638, Jun. 2019, doi: 10.1007/s11440-018-0758-y.

[2] ‘Effect of Temperature, pH, and Reaction Duration on Microbially Induced Calcite Precipitation’. Accessed: Dec. 02, 2025. [Online]. Available: <https://www.mdpi.com/2076-3417/8/8/1277>

[3] Y. Wang, Y. Wang, K. Soga, J. T. DeJong, and A. J. Kabla, ‘Microscale investigations of temperature-dependent microbially induced carbonate precipitation (MICP) in the temperature range 4–50 °C’, Acta Geotech., vol. 18, no. 4, pp. 2239–2261, Apr. 2023, doi: 10.1007/s11440-022-01664-9.

[4] C. Baley, M. Lan, A. Bourmaud, and A. Le Duigou, ‘Compressive and tensile behaviour of unidirectional composites reinforced by natural fibres: Influence of fibres (flax and jute), matrix and fibre volume fraction’, Mater. Today Commun., vol. 16, pp. 300–306, Sep. 2018, doi: 10.1016/j.mtcomm.2018.07.003.

[5] H. L. Bos, K. Molenveld, W. Teunissen, A. M. van Wingerde, and D. R. V. van Delft, ‘Compressive behaviour of unidirectional flax fibre reinforced composites’, J. Mater. Sci., vol. 39, pp. 2159–2168, Mar. 2004, doi: 10.1023/B:JMSC.0000017779.08041.49.

[6] S. Liang, P.-B. Gning, and L. Guillaumat, “Quasi-static behaviour and damage assessment of flax/epoxy composites,” Materials & Design, vol. 67, pp. 344–353, Feb. 2015, doi: 10.1016/j.matdes.2014.11.048.

[7] A. W. Van Vuure, J. Baets, K. Wouters, and K. Hendrickx, ‘Compressive properties of natural fibre composites’, Mater. Lett., vol. 149, pp. 138–140, Jun. 2015, doi: 10.1016/j.matlet.2015.01.158.

[8] R. Koh and B. Madsen, ‘Strength failure criteria analysis for a flax fibre reinforced composite’, Mech. Mater., vol. 124, pp. 26–32, Sep. 2018, doi: 10.1016/j.mechmat.2018.05.005.

[9] S. H. Vattathurvalappil, M. M. Shaukat, R. Theravalappil, S. Z. Shuja, and W. G. Abdelrahman,
‘Techno-environmental evaluation of alkaline treatment in flax reinforced thermoplastics’,
Polymers, vol. 16, no. 5, p. 662, Feb. 2024, doi: 10.3390/polym16050662.

[10] CarbonCloud, “Yeast extract (powder), EU – climate footprint,” [Online]. Available:
<https://apps.carboncloud.com/climatehub/product-reports/id/40832581969>

[11] CarbonCloud, “Ammonium chloride – climate footprint,” [Online]. Available:
<https://apps.carboncloud.com/climatehub/product-reports/id/77892067092>

[12] CarbonCloud, “Calcium chloride (CaCl₂), E509 – climate footprint,” [Online]. Available:
https://apps.carboncloud.com/climatehub/product-reports/id/146255465878

[13] Yara International, “Urea carbon footprint verification statement (DNV),” 2020. [Online]. Available:
https://ureaknowhow.com/wp-content/uploads/attachments/Urea%20Footprint.pdf

[14] Bcomp Ltd., “ampliTex™ 5009 technical datasheet,” 2021. [Online]. Available:
https://www.skibaumarkt.de/wp-content/uploads/Bcomp_amplitex_5009_en.pdf

[15] V. Kočí and T. Loubal,
‘LCA of liquid epoxy resin produced based on propylene and on glycerine’,
*Acta Environmentalica Universitatis Comenianae*, vol. 20, suppl. 1, pp. 62–67, 2012.
[Online]. Available: <https://fns.uniba.sk/fileadmin/prif/actaenvi/ActaEnvi_2012_Suppl.1/10_S_Koci_Loubal_Acta2012_Suppl_1.pdf>

[16] Sustainability Atlas,
‘Water-energy nexus optimization KPIs by sector (with ranges)’, Mar. 2026. [Online]. Available: <https://sustainableatlas.org/post/water-energy-nexus-optimization-kpis-by-sector-with-ranges-3242>

[17] Milieubarometer, “CO₂ parameters,” 2026. [Online]. Available: https://www.milieubarometer.nl/en-gb/carbon-footprint/co2-parameters/

[18] Thermo Fisher Scientific, “Heratherm compact incubator documentation,” [Online]. Available:
https://documents.thermofisher.com/TFS-Assets/LED/brochures/BRCTHTINCNA-0220.pdf

[19] Thermo Fisher Scientific, “Vacuum oven manual,” [Online]. Available:
https://documents.thermofisher.com/TFS-Assets/LSG/manuals/D21649~.pdf

[20] IKA, “C-MAG HS 7 technical data,” [Online]. Available:
<https://www.ika.com/en/Products-LabEq/Magnetic-Stirrers-pg188/C-MAG-HS-7-3581200/Technical-Data-cptd.html>

[21] LabTech S.r.l., “Vacuum pump product specifications,” [Online]. Available:
https://www.labtechus.com/product-page/vp30-vacuum-pump

[22] Mates Italiana S.r.l., “Peel ply 85 g/m² datasheet,” [Online]. Available:
https://fileserver.mates.it/Prodotti/5_Ausiliari/TDS/Peelply/PEELPLY85_DS.pdf

[23] R&G Faserverbundwerkstoffe GmbH, “Infusion mesh 160 g/m²,” [Online]. Available<https://www.r-g.de/en/art/FMC-39160200>

[24] Vacuum bagging film datasheet, “55 μm film,” [Online]. Available:
<https://5.imimg.com/data5/LB/MJ/ZF/SELLER-13421774/vacuum-bagging-film.pdf>
